# Letter to the Editor: Clarifying interpretation of cancer detection utility from whole-body MRI

**DOI:** 10.1007/s00330-025-12192-x

**Published:** 2025-12-05

**Authors:** Yosef Chodakiewitz, Daniel J. Durand, Alex Exuzides

**Affiliations:** Prenuvo Medical Group, Redwood City, CA USA

Dear Editor,

We read with great interest the systematic review and meta-analysis by Martins da Fonseca et al, evaluating whole-body MRI (WB-MRI) for opportunistic cancer detection in asymptomatic individuals [1]. As investigators in this area—and co-authors of one of the included cohorts (Westgate et al, cited as an AACR abstract [2])—we would like to offer clarifications that may help refine the interpretation of this important work.

The authors report a pooled confirmed cancer detection rate of 1.57% (95% CI 1.22–2.03%), which they describe as “modest” [1]. We suggest that qualitative descriptors are less informative than quantitative benchmarks. Yields for established imaging-based screening programs typically fall in the sub-percent to low-percent range: mammography detects cancers in approximately 0.4–0.5% of screens [3], and low-dose CT for lung cancer screening among high-risk smokers detects ~0.8–1.1% [4, 5]. In this context, a WB-MRI detection rate between 1.6% and 2.2% is at least comparable, and in some cases superior, underscoring the need for precise language when interpreting these findings.

It is also notable that our study (Westgate et al), one of the largest cohorts in the meta-analysis, was represented only at the abstract level, which necessarily limited interpretation [1]. The abstract itself reported the primary findings—including an overall cancer detection percentage of 2.2% (95% CI 1.37–3.28%), 64% of cancers detected at a localized stage, 68% occurring in organs without established single-cancer screening pathways, and two interval breast cancers (0.2%) identified during 12–16 months of follow-up. However, the complete analysis of this cohort—currently in pre-publication form and not yet peer-reviewed—provides the additional methodological detail and nuance needed to contextualize these results. This includes elaboration on cohort characteristics, potential sources of bias, nuances in patient-reported follow-up, and detailing of an observed ~99.8% negative predictive value for no interval cancer among individuals who did not undergo biopsy after WB-MRI (a proxy metric rather than a formal diagnostic NPV) [2]. These aspects, not appreciable from an abstract citation, are important for evaluating both the strengths and limitations of WB-MRI as a preventive screening tool.

The discussion of incidental findings and cost-effectiveness also warrants caution. Both are crucial issues, but neither was formally assessed in the included studies, and management strategies varied [1]. Future analyses should also incorporate rigorous health-economic modeling, accounting not only for the immediate costs of follow-up but also for downstream effects such as earlier-stage treatment, potential survival benefits, and associated cost savings. We support further research in these areas and recommend that conclusions clearly distinguish between evidence and hypothesis.

Both the review and our cohort emphasize the importance of standardized acquisition and reporting, such as ONCO-RADS, which serves not only as a reporting framework but also as an essential foundation for multi-center trials and data pooling. Furthermore, large-scale efforts such as the ongoing HERCULES Project, a prospective real-world observational study, will be critical in capturing diagnostic pathways and long-term outcomes [1, 2, 6]. Focusing on these priorities, rather than on qualitative labels, will best advance the field.

In sum, we do not dispute the pooled results but encourage refinements in framing. By situating WB-MRI yields alongside established screening benchmarks and by considering the fuller data from large cohorts, the modality’s potential value can be conveyed more accurately. Importantly, WB-MRI should not be viewed solely in comparison to single-cancer screening approaches such as mammography or low-dose CT, which are designed for narrowly defined populations and tumor types. Nor should it be positioned as a competitor to these targeted strategies. Rather, its role lies within the broader paradigm of multi-cancer detection (MCD), where the goal is to address the large proportion of cancers that currently lack dedicated screening pathways. For example, analysis of the National Lung Screening Trial demonstrated that nearly two-thirds of first primary cancers and almost half of cancer deaths in heavy smokers were attributable to non-lung cancers, underscoring the limitations of single-cancer screening and the opportunity for multi-cancer early detection strategies [7]. From a research perspective, WB-MRI should also be considered alongside emerging multi-cancer detection modalities such as blood-based assays, which interrogate complementary biological signals. Comparative effectiveness studies and hybrid strategies may ultimately determine the optimal role of each approach within a broader multi-omic framework. Recognizing this distinction in strategy helps clarify both the opportunities and the challenges of WB-MRI in preventive health. We appreciate the authors’ contribution and share their call for rigorous prospective evaluation to determine clinical utility, cost-effectiveness, and standardized pathways.

Sincerely,

Yosef Chodakiewitz, MD; Daniel J. Durand, MD; Alex Exuzides, PhD
